# Dangerous Worldview and Perceived Sociopolitical Control: Two Mechanisms to Understand Trust in Authoritarian Political Leaders in Economically Threatening Contexts

**DOI:** 10.3389/fpsyg.2021.603116

**Published:** 2021-03-25

**Authors:** Laura C. Torres-Vega, Josefa Ruiz, Miguel Moya

**Affiliations:** Department of Social Psychology, Faculty of Psychology, Mind, Brain and Behavior Research Center (CIMCYC), University of Granada, Granada, Spain

**Keywords:** authoritarianism, authoritarian political leader, economic crisis, socioeconomic status, economic threat, dangerous worldview, perceived sociopolitical control

## Abstract

In this research we analyzed the relationship between threatening economic contexts (i.e., undergoing the economic crisis and having low socioeconomic status) and trust in authoritarian ideologies and leaders, regardless of the left–right political axis. Based on two theoretical approaches (i.e., the dual-process model and the compensatory control model), we argue that this relationship is mediated by dangerous worldview and low perceived sociopolitical control. We conducted two correlational studies with samples of the general population. In Study 1 (*N* = 185), we found that perceived threat from the economic crisis and low socioeconomic status were correlated with a higher dangerous worldview, which resulted in a more authoritarian ideology (i.e., authoritarianism) and finally in greater trust in an authoritarian political leader. In Study 2 (*N* = 413), we replicated the findings of Study 1 and demonstrated that low perceived sociopolitical control was associated with higher authoritarianism. Moreover, low perceived sociopolitical control partially mediated the relationship between dangerous worldview and authoritarianism. Overall, our results show that two economically threatening contexts (i.e., the economic crisis and low socioeconomic status) promote authoritarianism and trust in authoritarian leaders (with unspecified political orientation) through psychological processes (i.e., perception of the social world and perceived control). These results are useful to understand and combat the rise of authoritarianism in our societies during financially difficult times such as economic crises.

## Introduction

Traditionally, the influence of macrosocial variables on the psychology of individuals has been the subject of few studies. Yet, in recent years, new lines of research in which both types of variables are explored have emerged in Social Psychology (see Moya and Fiske, 2017). Special interest has been given to negative macrosocial circumstances (e.g., wars, terrorist attacks, economic crises, and poverty) and how they affect the psychology of ordinary people. In this research, we focused on the last two variables: belonging to a disadvantaged social class and undergoing the effects of the economic crisis. Both circumstances imply an economic threat to people, with all its associated factors and consequences. However, they differ in the following: economic crises are temporary by definition and affect all social classes to a greater or lesser extent, but social class tends to be relatively stable in individuals; despite beliefs to the contrary, reality shows that the possibility of changing to another social class in Western countries is low (Moya and Fiske, 2017). We were interested in exploring how these two situations influence people’s adherence to authoritarian ideologies (hereinafter authoritarianism) and preference for political leaders with an authoritarian leadership style, considering the first variable as a direct antecedent of the second.

In recent years, the level of political and social authoritarianism in Western countries has become a concerning and significant issue. In fact, a study conducted by YouGov in 2016 revealed that the percentage of voters with populist authoritarian views—rejection of immigration, preference for a hard foreign policy and opposition to human rights laws, the institutions of the European Union and European integration policies—in Romania, Poland and France was higher than 60% (Twyman, 2016). This percentage was lower in Spain (33%), but the findings also suggest a considerable level of authoritarian attitudes in Spanish society (Twyman, 2016).

Apart from this apparent increased support for authoritarian ideologies, the rise and/or consolidation of political parties and leaders with a marked authoritarian leadership style has been particularly concerning in recent years. Such is the case of far-right parties in various European countries such as Denmark, France, Switzerland, Poland, Hungary, and Austria (Statista, 2019). The growing support of parties with these political views has also been observed in other European countries, although to a lesser extent. Some examples are Italy—where the Lega Nord obtained 17.4% of the votes in the latest general elections held in 2018—and Germany—where Alternative für Deutschland obtained 12.6% of the votes in 2017—(Statista, 2019). The rise of far-right parties and their leaders is also happening in Spain, where VOX obtained 10.97% of the votes in the latest elections to the Parliament of Andalusia, one of its Autonomous Communities or regions (El País, 2018) and 10.3% of the votes in the elections to the Spanish Parliament held on April 28, 2019 (El País, 2019). Thus, the far right stormed into the Spanish political sphere, something unprecedented until then.

Authoritarianism and authoritarian leadership styles are also present in activists from far-left groups and parties (Van Hiel et al., 2006). The Venezuelan regime is an example of a left-wing authoritarian leadership and some media consider the Movimento 5 Stelle (Italy)—a party that defines itself ideologically as “neither left nor right”—as an “authoritarian organization” (Caruso, 2017).

### Authoritarianism and Authoritarian Leaders: Concept and Measure

The study of authoritarianism has a long tradition in the social sciences, dating from the pioneering works of Reich (1933); Horkheimer et al. (1936), and, especially, the work of Adorno et al. (1950) on the authoritarian personality. However, research on authoritarianism has faced three major problems that, at present, continue to be subject to debate in the specialist literature. The first of them has to do with the conceptualization of the construct itself. Authoritarianism has traditionally been understood as a personality variable, with Altemeyer’s Right-Wing Authoritarianism (RWA) theory being the most popular within this approach (Altemeyer, 1981, 1988, 1996). According to it, authoritarianism is a construct composed of three dimensions: (a) Authoritarian Aggression—the intention to hurt another person or group physically or psychologically based on the belief that the aggression is sanctioned by the authorities or necessary to protect authority—; (b) Authoritarian Submission—a general acceptance of what is said and done by those in a position of authority and the willingness to obey the authorities without questioning them—; and (c) conventionalism—strong acceptance of and adherence to traditional social norms. However, the difficulties that authoritarian personality theories have encountered in attempting to predict authoritarian behavior, together with evidence showing that behaviors normally considered authoritarian can be induced by situational factors, have motivated the development of new conceptualizations of authoritarianism (Oesterreich, 2005). For example, Feldman (2003) understands authoritarianism as a reflection of the tension between the social values of autonomy, or individual freedom, and social conformity, which would be intensified under situations of threat to the social order. Similarly, Duckitt (2001) considers authoritarianism a motivational response directed toward maintaining order and social stability, and posits that social conformity together with exposure to a threatening context would favor such authoritarian motivation (we will develop this perspective in the next section). Authoritarianism has also been conceptualized as a reaction to threatening situations in which the individual feels that they do not have the cognitive, emotional, and social resources to deal with the situation (Oesterreich, 2005). This authoritarian reaction could be relatively infrequent, such as when the individual is faced with a highly threatening and stressful situation, but it could also shape a trend toward authoritarianism (i.e., authoritarian personality) in those individuals who have learned from childhood that they do not have the necessary skills and resources to face difficult situations (Oesterreich, 2005). Although each of these approaches has its particularities, they share two fundamental ideas: (a) authoritarianism is mainly an individual response to threatening situations, and (b) although there may be a certain predisposition to authoritarianism in some people, contexts that threaten security and social order can favor authoritarian attitudes in any individual.

The latter connects with the second major problem of research into authoritarianism: its link with the conservative political orientation. There is still no consensus in the specialist literature about whether authoritarianism is only observed in people with a conservative ideology (e.g., Stone, 1980; Stone and Smith, 1993; Altemeyer, 1996; Jost et al., 2003) or can also be seen in people with a left-wing ideology (Eysenck, 1954; Ray, 1983; McFarland et al., 1996; Feldman, 2003; Mullen et al., 2003; Van Hiel et al., 2006; Conway et al., 2018). Based on the more situational perspectives of authoritarianism (e.g., Duckitt, 2001; Feldman, 2003; Oesterreich, 2005; Stellmacher and Petzel, 2005), we consider that people on both the left and the right can show authoritarian attitudes in response to a threatening situation.

Assuming this perspective leads us to confront the third pitfall in the study of authoritarianism: how it may be measured. Although Altemeyer’s Right-Wing Authoritarianism (RWA) Scale is perhaps the most popular measure of authoritarianism, it has also received a great deal of criticism because many of its items refer to attitudes and behaviors typical of right-wing people. Therefore, the RWA Scale prevents the separation of authoritarianism from right-wing political orientation and it overlaps the authoritarian construct with the behaviors and attitudes that it seeks to predict (Feldman, 2003; Oesterreich, 2005; Stenner, 2005). Recently, Dunwoody and Funke (2016) have developed the Aggression-Submission-Conventionalism Scale (ASC), a three-factor scale based on the three dimensions of authoritarianism proposed by Altemeyer, but generating new items not linked to right-wing attitudes and behaviors. Thus, in our research, in order to measure authoritarianism independently from political orientation, we will use the ASC scale, although we will not consider the sub-dimension of conventionalism. According to Dunwoody and Funke (2016), conventionalism is the factor most associated with right-wing political orientation and is hence not a necessary component of authoritarianism *per se*; by contrast, authoritarian aggression is the most consistent and powerful component of the construct, followed by authoritarian submission (Dunwoody and Funke, 2016). In fact, authors such as Van Hiel et al. (2006) only consider authoritarian aggression and submission when measuring left-wing authoritarianism. In our research, we used the same strategy as Van Hiel et al. (2006) to measure authoritarianism in the Spanish context, regardless of political orientation.

We also intended to explore the preference for authoritarian political leaders regardless of their political orientation, focusing on their leadership style. In the literature on leadership styles in the organizational context (e.g., Bass and Bass, 2008), authoritarian leaders are described as being strong and directive, with four general characteristics: (a) they make all the important decisions; (b) they are more committed to fulfilling their tasks and obligations than concerned about the well-being of their subordinates; (c) they keep a considerable social distance from their subordinates; and (d) they motivate their subordinates mainly through punishments and threats. In our research we explored trust in political leaders with an authoritarian style regardless of the left–right ideological axis.

### Antecedents of Authoritarianism and the Acceptance of Authoritarian Leaders

On the basis of the recent conceptualizations of authoritarianism mentioned above (i.e., Duckitt, 2001; Oesterreich, 2005), in this study, we will investigate two variables that may favor authoritarianism as a response to threatening situations: dangerous worldview and perceived control.

First, according to the dual-process model of ideology and prejudice (Duckitt, 2001), authoritarianism is conceptualized as a social or ideological attitude which express the motivational goal of social control and security. The model postulates that authoritarianism is promoted by a view of the world as an essentially insecure and dangerous place (e.g., Sibley and Duckitt, 2013). Dangerous worldview results from the combination of a personality high in social conformity and the socialization in and exposure to threatening contexts; hence it is thought to be relatively stable over time (Duckitt and Fisher, 2003). We argue that having a disadvantaged socioeconomic status can favor a dangerous worldview, since people with low SES grow up in neighborhoods in which insecurity is fostered by high levels of unemployment (Pan Ké Shon, 2012). Unemployment is also one of the main indicators of economic crisis, so one might think that perceived threat from a crisis affects dangerous worldview. In other words, although dangerous worldview is a relatively stable variable, it is also a reflection of social reality; thus, when social reality changes drastically and becomes more insecure (for example, with the sharp fall in employment in the context of economic crisis), our worldview is also likely to change (Duckitt and Fisher, 2003). The relationship between dangerous worldview and (right-wing) authoritarianism has received empirical support from various studies—see the meta-analysis by Perry et al. (2013) and the longitudinal study by Sibley and Duckitt (2013). As regards trust in authoritarian political leaders, most studies on authoritarianism postulate that authoritarian leaderships tend to emerge under negative or highly uncertain circumstances, in which strong and dominant leaders are perceived as the solution to the problem (Rast et al., 2013; Harms et al., 2018). Similarly, Sprong et al. (2019) found that the feeling of anomie—the socially shared perception that it is not possible to trust others and that people are not guided by moral principles—, a similar concept to dangerous worldview, partially mediated the relationship between an economically hostile context and the wish for a strong and dominant leader.

Second, as regards perceived control, the literature shows that individuals have developed a basic motivation to defend themselves from the perception that the surrounding world is random and chaotic (e.g., Heider, 1958). In other words, individuals need to feel that the environment is predictable and controllable. Based on this premise, the compensatory control model (e.g., Kay et al., 2008, 2009) postulates the following: when individuals perceive a lack of control—either chronic or not—over their environment, they are likely to adopt ideologies (e.g., authoritarianism) that allow them to regain the feeling that their environment is structured and predictable and increase perceived personal, social or religious control (Landau et al., 2015). Previous studies have shown that low perceived control mediated the relationship between perceived social threat and increased authoritarianism among participants with previous low scores on authoritarianism (Mirisola et al., 2014). Lack of control constitutes an underlying point of argument in different analyses on the rise of authoritarian leaders (e.g., Fromm, 1941; Arendt, 1958; Oesterreich, 2005; Teymoori et al., 2017). We took this literature as our basis for empirically analyzing the link between perceived control, on the one hand, and authoritarianism and trust in an authoritarian political leader (independently of his/her political orientation), on the other. In addition to this, according to the compensatory control model, people can delegate the function of control to the government as an external system to reestablish the structure and order of their social world (e.g., Kay et al., 2008; Landau et al., 2015). For example, it has been reported that the decrease of perceived control increases the preference for governmental control and support for the incumbent government (Kay et al., 2008).

Most of these studies have analyzed the role of loss of personal control in the search for external control sources (e.g., authoritarian ideology and governmental control). Yet, other theoretical approaches (e.g., group-based control model; Fritsche et al., 2013) argue that, when individuals feel that their personal control is being threatened, they try to reestablish it first of all through their feeling of social control, for example, by identifying with groups perceived as being agentic (i.e., social self; Fritsche et al., 2013). If they do not manage to recover the feeling of control through the social self, they may use compensatory control strategies that reaffirm the feeling of order and structure in the world (Fritsche and Jugert, 2017), for example by preferring hierarchies or supporting the government (Landau et al., 2015). In our research we intended to analyze the role of individuals’ perceived influence or control over what happens in their sociopolitical sphere (Paulhus and Christie, 1981). We consider that this dimension of perceived control is relevant for two reasons: (a) it allows us to explore the relationships between the different variables in the same context (i.e., the social and political context) and (b) this dimension of control is closer to the concept of social self (Fritsche et al., 2013) because it reveals to what extent individuals think that citizens can influence their social and political context.

### Economic Crisis and Trust in Authoritarian Ideologies and Leaders

The rise of authoritarian parties and leaders has coincided in time with the global economic crisis that began in 2008 and whose consequences are still present in many European and non-European countries (United Nations, 2019). The economic crisis can be understood as a clearly threatening situation that determines our perception of the world around us (i.e., dangerous worldview); this in turn can influence individuals’ feeling of control. Focusing on the case of Spain, we consider that this influence may have been mainly due to two characteristics of the crisis: socioeconomic threat and uncertainty.

First of all, the crisis implies a threat in terms of economic and social well-being. In Spain, while the situation seems to have improved the macroeconomic level—a recovery that will probably be interrupted by the Covid-19 pandemic—, many Spanish households are still harshly suffering from the crisis. According to data from the Survey on Living Conditions published by the Spanish National Statistics Institute (INE, Instituto Nacional de Estadística, 2018), in 2017 the rate of poverty risk was 21.6% (even higher than 19.8% in 2008, before the onset of the crisis), 8.3% of households experienced energy poverty, 37.3% of Spanish families could not face unexpected expenses and 34.4% could not afford to go on holiday for at least a week. As regards unemployment, the data provided by INE on the third quarter of 2019 (Instituto Nacional de Estadística, 2019) showed a 13.92% unemployment rate, which mainly affects young people under 25 (the unemployment rate in this age group is 33%, the second highest rate behind Greece in the European Union; Statista, 2020). The crisis has also led to growing inequality, even more in Spain than in other countries also affected by the crisis. Spain is the fifth most unequal country in the European Union, along with Italy (Eurostat, 2021a). In fact, the Gini index rose in Spain from 32.4 in 2008 to 33.2 in 2018 (Eurostat, 2021b). Previous studies have shown that this type of socioeconomic indicators (e.g., unemployment, low income level) is associated with a greater respect for authority and obedience among the population (i.e., greater authoritarianism; Onraet et al., 2013). Moreover, this context of economic decadence erodes the trust of citizens in politics and institutions (e.g., Roth, 2009; Ervasti et al., 2019; Tormos, 2019). Citizens can feel attracted to new political options that are different from traditional ones—and sometimes radical and antidemocratic (Werts et al., 2013)—and advertised as the only possible solution through a colloquial, emotional, simple and direct language that is hard with opponents (Moffit and Tormey, 2014). Previous studies have also shown that economically hostile contexts increase people’s preference for authoritarian political leaders that ignore the existing political parties (Krieckhaus et al., 2014) and break the rules (Sprong et al., 2019).

Second, the economic crisis has also dramatically changed people’s perception of the stability of the world around them, which is no longer considered as predictable and controllable (Jetten et al., 2017). For example, Rosenthal et al. (1989, p. 10) consider that a crisis context can be understood as “a serious threat to the basic structures or the fundamental values and norms of a social system, which under time pressure and highly uncertain circumstances necessitates making vital decisions.” The literature shows that experiencing terrorist attacks—another type of social threat with a high component of uncertainty about the future—has been associated with greater adherence to authoritarian ideologies (Moya and Morales-Marente, 2005; Bonanno and Jost, 2006; Echebarria-Echabe and Fernández-Guede, 2006). However, it should be noted that most research in this field has focused on the relationship between threatening situations and authoritarianism understood as a right-wing or conservative policy. Authoritarian leadership style generally has negative connotations and generates some aversion. Yet, it seems that, in times of crisis and uncertainty when unpopular decisions must be taken for the situation to change, authoritarian leaderships are perceived as necessary (Bass and Bass, 2008). Specifically, it has been reported that voters valued a strong and directive leadership more positively in a terrorist attack condition compared to a social well-being condition (Merolla and Zechmeister, 2009). Other studies have also found that male faces and deep voices (i.e., dominant traits) are better rated in contexts of war and intergroup conflict (compared to contexts of peace and cooperation; e.g., Laustsen and Petersen, 2017).

Based on the above, we considered that these two characteristics of the economic crisis (i.e., socioeconomic threat and uncertainty) were likely to promote endorsement of authoritarianism and authoritarian political leaders through the feeling that the social environment is insecure and threatening (i.e., dangerous worldview) and uncontrollable (i.e., low perceived sociopolitical control).

### Social Class and Trust in Authoritarian Ideologies and Leaders

Social class, also known as socioeconomic status (SES), refers to a social stratification system based on access to resources such as wealth, education and prestige (Kraus et al., 2012; Stephens et al., 2012). Numerous studies have shown that SES influences the cognition, emotion and behavior of individuals (for a review, see Manstead, 2018; see also Piff et al., 2018). We considered that belonging to a low social class had common characteristics with those previously attributed to the economic crisis: socioeconomic threat and uncertainty. Thus, in our research we aimed to analyze whether SES is associated with trust in authoritarian ideologies and leaders in a similar way as that reported for threat from the economic crisis, that is, through dangerous worldview and low perceived sociopolitical control.

As regards the relationship between SES and dangerous worldview, previous studies have shown that people with low SES are more sensitive and/or attentive to threats than people with high SES (see Kraus et al., 2012). Moreover, Chen and Matthews (2001) found that children belonging to a low social class showed a higher heart rate and blood pressure after being exposed to an ambiguous social threat scenario and perceived a greater threat in videos that reflected hostile interactions ambiguously.

Conversely, this greater predisposition to perceive threats in the environment (i.e., a higher dangerous worldview) may influence the feeling of control of individuals with low SES. People who grow up in a middle/high social class are likely to perceive environmental difficulties as challenges that they can overcome (i.e., that they can control). By contrast, people who grow up in a working-class environment are likely to perceive these difficulties as threats that they must avoid (i.e., that they cannot control) (Manstead, 2018). These differences in the way of coping with challenges can be explained by the differences in social and cultural capital between the social classes, which influence how people who grow up in each of these contexts build themselves and their social environment (Manstead, 2018). The very limited sense of control experienced by working-class people is also being accentuated by high levels of unemployment and the flexibilization of the labor market (the easing of conditions for redundancy, part-time and/or temporary contracts, etc.) that preclude the possibility of a solid and stable livelihood on which to build a life project (Bauman, 2013). Furthermore, our society’s tendency toward individualism also undermines the sense of control of low-status people: being unemployed or having a precarious and unstable job is interpreted on an individual basis as an inability on the part of working-class people to opt for something better, because they do not possess the necessary skills and qualifications (Bauman, 2013). There is empirical evidence that participants who perceive themselves as belonging to a low (vs. high) social class report lower perceived control over the events that occur in their lives (Kraus et al., 2009). It has also been found that perceived control is a key aspect to explain the class differences in behaviors related to political participation, such as the support for pro-environmental actions (Eom et al., 2018). Similarly, individuals’ socioeconomic status has been positively associated with political participation, and perceived political efficacy—which is greater in individuals with high SES—is the mechanism that underlies this relationship (Krauss, 2015).

Regarding the relationship between SES and trust in authoritarian ideologies and leaders, Carvacho et al. (2013) found that the lower the income and educational level of individuals—two of the usual indicators to measure objective social class—, the higher their scores on authoritarianism. Moreover, Tuticì and von Hermanni (2018) found that low-SES participants, unemployed persons and people who perceived greater deprivation in socioeconomic terms tended to support the far-right party Alternative für Deutschland more than high-SES participants. In Spain, this negative relationship between SES and support for authoritarian parties seems less clear. Although the voters of VOX—a far-right authoritarian party—in the general elections held in April 2019 were mainly those with medium and high income (Aumaitre, 2019), the results of the latest general elections held in November 2019 suggest that the relationship between income level and voting for VOX is more complex, as greater support for this party was found both in rich districts and poor cities (Andrino et al., 2019). As regards the preference for authoritarian political leaders among people of different social classes, we are not aware that any empirical studies have explored this aspect; yet, we agree with the approach of Brown-Iannuzzi et al. (2017), who reported that the lower perceived control of people with low SES leads them to seek external sources of control, such as a strong and directive government. Furthermore, as mentioned above, the absence of a project for the future (as a consequence of unemployment and labor flexibility), together with the globalization of the economy—which moves power from local politicians to other global agencies (Bauman, 2013)—can contribute to working-class people perceiving politicians who have traditionally held their support as incapable of improving their living conditions and, thereby becoming attracted to new figures who promise to take control of the situation.

### The Present Research

**FIGURE 1:**
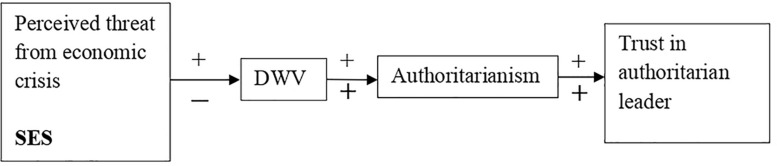
Proposed model for the relationship between the macrosocial variables (economic crisis and SES) and trust in an authoritarian political leader, through dangerous worldview (DWV) and authoritarianism.

Our aim was to explore the influence of two macrosocial variables (i.e., economic crisis and social class—SES) on adherence to authoritarianism and thus on the preference for political leaders with an authoritarian leadership style. We assumed that both economic crisis and belonging to a low social class are threatening situations for individuals, promoting their perception of the social world as insecure, unpredictable, and dangerous; this dangerous worldview, in turn, can decrease individuals’ perceived control of the social context. In our research, we intended to explore the effect of these variables on authoritarianism without regard to political orientation. We conducted two studies. Study 1 aimed to analyze whether feeling threatened by the economic crisis and belonging to a low social class contribute to viewing the world as a dangerous place, and whether this view is in turn associated with a more authoritarian ideology, as proposed by the dual-process model (Duckitt, 2001). In Study 1 we took a further step by focusing on trust in an authoritarian leader (without specifying whether the ideology was left- or right-wing) as the ultimate consequence of the increase in dangerous worldview and authoritarian ideology (see Figure 1). We hypothesized that both perceived threat from the economic crisis (Hypothesis 1) and belonging to a low social class (Hypothesis 2) would be associated with greater trust in an authoritarian leader through dangerous worldview and authoritarian ideology (in this order).

In Study 2 we analyzed whether perceived lack of control leads to an increase of authoritarian ideology as a compensatory control mechanism (e.g., Kay et al., 2008). Previous studies have explored the relationship between personal control—a dimension of control that is close to self-efficacy—and authoritarianism. By contrast, in our research we explored sociopolitical control, understood as the feeling that citizens have an influence on the social and political issues of their environment. Thus, in Study 2 we expected to find that, as observed for personal control (e.g., Mirisola et al., 2014), lack of sociopolitical control was associated with greater authoritarianism (Hypothesis 3). In this study, we also explored whether dangerous worldview was associated with higher authoritarianism through lower perceived sociopolitical control, although this mediation was not pre-registered, even as an exploratory line of investigation.

Both studies consisted of cross-sectional surveys. We measured trust in an authoritarian leader (and the remaining variables) with questionnaires. In both studies, the target was a leader with an authoritarian style but unspecified political orientation (i.e., no reference was made to the leader’s ideology). Our research was aimed at making a double contribution to knowledge in the area of political psychology by: (a) analyzing how two hostile socioeconomic contexts may influence sociopolitical attitudes (i.e., authoritarian ideology and trust in authoritarian political leaders) through psychological processes (i.e., perception of the social world and feeling of control); and (b) trying to avoid the left–right ideological bias when exploring these processes, both in the authoritarianism scale and in the measure of trust in an authoritarian leader.

## Study 1

### Materials and Methods

Supplementary Material, dataset, and syntax appertaining to Study 1 are available at the following link: https://osf.io/pm8u4/?view_only=81c56cebeb2f43a4b7d4f3dae1907329

#### Participants

A total of 310 people from the general population accepted to complete the questionnaire. However, we applied a selection procedure to rule out participants who were not Spanish, had not completed the total questionnaire or had not paid attention to the questions (see Supplementary Material and Supplementary Table 1 for more details concerning the exclusion procedure for participants). The final sample was composed of 185 participants (86 women) with ages ranging from 18 to 67 years (*M* = 43.39; *SD* = 12.10). Information on the socioeconomic status (i.e., educational and income level) of participants is shown on Table 1.

**TABLE 1:** Distribution of the participants socioeconomic status (educational and income levels) in Studies 1 and 2.

Variable	Study 1		Study 2	
		
	*n*	%	*n*	%
**Family income**				
<1,000€	30	16.6	61	14.8
1,000€–2,000€	82	44.3	157	38
2,000€–3,000€	37	20.4	103	24.9
3,000€–4,000€	14	7.7	44	10.9
4,000€–5,000€	7	3.9	24	5.8
>5,000€	11	6.1	13	3.1
Not reported	4	2.2	10	2.4
**Maternal education**				
Primary school	112	61.2	140	33.9
Secondary education/school graduate	30	16.4	78	18.9
Vocational training	10	5.5	41	9.9
High school/diploma	8	4.4	47	11.4
University not completed	4	2.2	12	2.9
University completed	19	10.4	92	22.3
Not reported	2	1.1	3	0.7
**Paternal education**				
Primary school	86	47	132	32
Secondary education/school graduate	32	17.5	86	20.8
Vocational training	21	11.5	46	11.1
High school/diploma	14	7.7	39	9.4
University not completed	4	2.2	15	3.6
University completed	26	14.2	91	22
Not reported	2	1.1	4	1
**Participant education**				
Primary school	7	3.8	14	3.4
Secondary education/school graduate	20	10.8	29	7
Vocational training	29	15.7	32	7.7
High school/diploma	17	9.2	52	12.6
University not completed	29	15.7	101	24.5
University completed	83	44.9	184	44.6
Not reported	0	–	1	0.2

#### Procedure

We developed an online questionnaire with the Qualtrics platform and distributed it through three different channels. We asked psychology students to send the link to the questionnaire to their relatives and offered them a small course credit reward in exchange. The two other distribution channels were flyers that included basic information on the questionnaire and a link to access it, which were circulated to people from the general population either physically (at the bus station of a city in southern Spain) or through the social media (mainly Facebook). The flyers advertised that respondents to the survey would enter a draw from which they could win a 32 GB Mini Retina iPad. Data were collected between March 05 and April 18, 2017. We also gave participants the email address of the person in charge of the study so that they could ask for more information. This research (Studies 1 and 2) is part of a research project that received the approval of the Ethics Committee of the University of Granada. Before participants started to answer the survey, they all gave their consent to participate voluntarily in the study, in accordance with the Declaration of Helsinki.

#### Measures

##### Perceived threat from the economic crisis

We adapted two items used by Becker et al. (2011) to Spanish: “To what extent do you feel threatened by the current economic situation in Spain?” and “To what extent have you personally been affected by the current economic crisis?” (from 1, *not at all*, to 5, *very much*). These two items were highly correlated with each other (*r* = 0.56) and the mean between both was taken as one single indicator of perceived threat from the economic crisis; higher scores indicated greater threat.

##### Socioeconomic status (SES)

We determined the objective social class of participants using a series of indicators based on the monthly income of the family (from 1, *less than 1,000€*, to 6, *more than 5,000€*) and the level of educational attainment of the father, the mother and the participant (from 1, *Primary school*, to 6, *University completed*) (see Table 1). We obtained a single indicator of the SES from the mean of the standardized scores of each indicator mentioned; higher scores indicated higher SES.

##### Dangerous worldview

We adapted the measure used by Duckitt et al. (2002), which is composed of 10 items that assess the social perception that the world is an insecure and unpredictable place. The measure includes items such as “My knowledge and experience tells me that the social world we live in is basically a dangerous and unpredictable place, in which good, decent and moral people’s values and way of life are threatened and disrupted by bad people.” It had a seven-point Likert response format (from 1, *strongly disagree*, to 7, *strongly agree*). We recoded items worded inversely so that higher scores reflected a greater perception that the world is a dangerous place (α = 0.76).

##### Authoritarianism

We used the Aggression-Submission-Conventionalism scale (ASC, Dunwoody and Funke, 2016), composed of 18 items. Six items measure Authoritarian Aggression (e.g., “Strong force is necessary against threatening groups”); six items measure Authoritarian Submission (e.g., “We should believe what our leaders tell us”); and six items measure Conventionalism (e.g., “Traditions are the foundation of a healthy society and should be respected”). The scale had a Likert-type response format ranging from 1(*strongly disagree*) to 5 (*strongly agree*). One of the advantages of this instrument compared to the Right-Wing Authoritarianism scale (Altemeyer, 1981) is that it identifies three separate (although related) factors of authoritarianism, which makes it possible to separate it from a right-wing political ideology (Dunwoody and Funke, 2016). In our two studies we only considered the Authoritarian Aggression and Authoritarian Submission subscales to develop our measure of authoritarianism, obtaining a single global score (α = 0.71). We recoded items worded inversely so that higher scores reflected higher levels of authoritarianism.

##### Trust in an authoritarian leader

We presented participants with a text that described an authoritarian leader (see the Supplementary Material). The description was based on items of the Autocratic Leader Behavior Scale (De Hoogh et al., 2004; see also De Hoogh and Den Hartog, 2009), which includes dominant behaviors and shows that authoritarian leaders are mainly concerned with protecting their own position, make decisions on their own without considering the suggestions of their subordinates, and usually give them orders about what they should do (e.g., “This leader makes decisions alone without asking for suggestions”). Once they read the description, participants had to respond to four items on trust in the leader adapted from Rast et al. (2013): (a) “I would absolutely trust this leader”; (b) “I think this leader would do the right thing for Spain to overcome the crisis”; (c) “This leader would be very committed to Spanish society”; (d) “This political leader would like the best for Spanish society.” The scale had a Likert-type response format (from 1, *strongly disagree*, to 7, *strongly agree*) and good internal consistency (α = 0.90). Given that there is grammatical gender in Spanish, we used gender-neutral wording.

Apart from these measures, participants were asked to report their age, sex and political orientation on a continuum from (1, *far left*, to 10, *far right*).

### Results

First, we performed a MANOVA, with all the variables explored as dependent variables in order to identify any significant differences in the variables, at baseline, among the three sample types (i.e., relatives of students, people recruited at the bus station, or recruited through social media). Results showed no statistically significant differences between the participants’ mean scores for each of the dependent variables (Supplementary Table 3); therefore, we performed the rest of the analyses by considering the full sample.

Secondly, we ran a two-tailed bivariate correlation analysis to identify the relationship between variables (Table 2). In line with Hypotheses 1 and 2, both perceived threat from the economic crisis (positively) and participant’s SES (negatively) correlated with dangerous worldview. Dangerous worldview positively correlated with authoritarianism, and authoritarianism positively correlated with trust in an authoritarian leader. All correlations were statistically significant and all correlation coefficients were higher than | *r* = 0.14|, which, according to the sensitivity test performed, was the minimum effect size that could be detected in our sample (*N* = 185, power = 0.80 and α = 0.05).

**TABLE 2:** Descriptive statistics (means and standard deviations) and bivariate correlations between the variables included in Study 1 (below the diagonal) and Study 2 (above the diagonal).

	1	2	3	4	5	6	7	8	9	10	*M* (Study 2)	*SD* (Study 2)
(1) Economic crisis (threat)	–	−0.21***	0.28***	0.05	–0.06	0.01	–0.05	0.10*	0.043	−0.129**	2.93	0.90
(2) SES	−0.16*	–	−0.32***	–0.00	−0.12*	–0.06	0.15**	0.10***	−0.23***	0.050	0.01	0.73
(3) Dangerous worldview	0.22**	−0.15*	–	−0.13**	0.20***	0.12*	0.06	−0.26***	−0.09†	−0.169**	3.87	0.99
(4) Sociopolitical control				–	−0.19***	−0.16**	−0.22***	0.24***	–0.08	–0.017	4.12	0.79
(5) Authoritarianism	–0.10	−0.13†	0.20**		–	0.31***	0.43***	−0.24***	0.13**	0.114*	2.25	0.56
(6) Trust in authoritarian leader	–0.04	−0.16*	0.19**		0.38***	–	0.15**	−0.16**	–0.04	0.179***	1.86	1.14
(7) Political orientation	–0.04	0.11	0.12		0.37***	0.16*	–	−0.10*	0.08	–0.038	4.28	1.62
(8) Political interest								–	0.11*	0.085	3.32	0.87
(9) Age	–0.04	−0.26***	–0.04		0.13†	–0.03	0.124		–	0.036	30.98	13.05
(10) Sex	–0.02	–0.053	–0.11		0.17*	0.04	–0.057		0.073			
*M* (Study 1)	3.56	–0.01	3.62		2.19	1.77	4.53		43.39			
*SD* (Study 1)	0.83	0.69	1.01		0.51	1.07	1.68		12.10			

^†^p < 0.066, *p < 0.05, **p < 0.01, ***p < 0.001.

**FIGURE 2:**
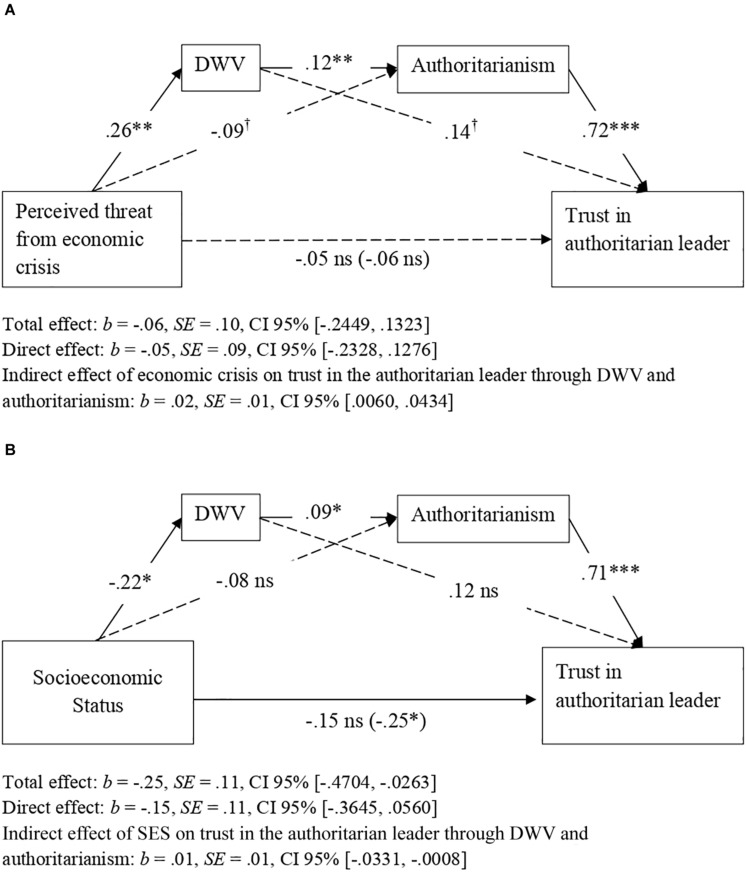
Indirect effects of perceived threat form economic crisis **(A)** and SES **(B)** on trust in an authoritarian political leader via dangerous worldview (DWV) and authoritarianism (Study 1). Figure shows the effects without consideration of covariates. Non-significant effects appear with dashed lines. Unstandardized coefficients presented. *p < 0.05, **p < 0.01, ***p < 0.001, ^†^*p* < 0.066.

To verify whether perceived threat from the economic crisis (Hypothesis 1) and participant SES (Hypothesis 2) influenced trust in an authoritarian political leader through higher dangerous worldview and higher authoritarian ideology, we tested two separate serial mediation models using the PROCESS macro for SPSS (Hayes, 2013; Model 6, bootstrapping procedure, 10,000 repeats and CI 95%).^1^ We took either the threat from the economic crisis or the participant’s SES as the predictor variable in each analysis. The remaining variables were used in the same way in both analyses: we introduced trust in an authoritarian leader as a criterion variable, dangerous worldview as a first mediator, and authoritarianism as a second mediator. As predicted by Hypothesis 1, threat from the economic crisis was associated with higher dangerous worldview, which in turn was associated with higher authoritarianism. Finally, authoritarianism was associated with greater trust in an authoritarian leader. Results showed that the threat from the economic crisis had not a total effect on trust in the leader, but there was a significant and positive indirect relationship between both variables through dangerous worldview and authoritarianism (Figure 2A). Results also supported Hypothesis 2, given that the lower the SES of participants, the higher their view of the world as a dangerous place; this was associated with a more authoritarian ideology, which in turn was associated with greater trust in an authoritarian leader (Figure 2B). In this case, there was a statistically significant total effect of participant SES on trust in the leader, but this effect was fully mediated by dangerous worldview and authoritarianism. More interestingly, the indirect effect of both threat from the economic crisis and participant SES on trust in an authoritarian leader, attributable to dangerous worldview and authoritarianism, remained statistically significant when the covariates and both predictors were included in the analysis (Table 3). Following the recent recommendations by Yzerbyt et al. (2018), to correctly test a mediation model and avoid reporting false positives, both the indirect effect and each of the paths of the indirect route must be statistically significant. This requirement is valid both for the effect of the economic crisis and for SES, as shown on Figures 2A,B and Table 3.

**TABLE 3:** Direct, indirect and total effect of threat by economic crisis and SES on trust in an authoritarian political leader including both predictors in the analysis and controlling by age, sex, political orientation (Studies 1 and 2) and political interest (Study 2).

	Study 1 (*N* = 185)						Study 2 (*N* = 413)					
		
	*B* (β)	(*SE*)	*t*	*p*-value	LL 95% CI	UL 95% CI	*B* (β)	(*SE*)	*t*	*p*-value	LL 95% CI	UL 95% CI
**Direct effects**												
Economic crisis on dangerous worldview	**0.23** (0.19)	(0.09)	2.61	0.010	0.0562	0.4056	**0.26** (0.24)	(0.05)	5.08	<0.001	0.1585	0.3589
Economic crisis on authoritarianism	−**0.08** (−0.14)	(0.04)	–2.01	0.046	–0.1666	–0.0014	−0.05 (0.09)	(0.03)	–1.70	0.090	–0.1057	0.0076
Economic crisis on trust in authoritarian leader	−0.08 (−0.07)	(0.09)	–0.91	0.363	–0.2661	0.0980	0.05 (0.05)	(0.06)	0.79	0.428	–0.0749	0.1761
SES on dangerous worldview	−**0.24** (−0.17)	(0.11)	–2.19	0.030	–0.4588	–0.0234	−**0.39** (−0.28)	(0.07)	–5.73	<0.001	–0.5174	–0.2530
SES on authoritarianism	−**0.10** (−0.14)	(0.05)	–2.01	0.046	–0.2066	–0.0018	−0.07 (−0.09)	(0.04)	–1.89	0.059	–0.1479	0.0029
SES on trust in authoritarian leader	−**0.23** (−0.15)	(0.11)	–2.02	0.045	–0.4564	–0.0051	0.03 (0.02)	(0.09)	0.31	0.753	–0.1404	0.1939
Dangerous worldview on authoritarianism	**0.09** (0.17)	(0.03)	2.70	0.008	0.0254	0.1627	**0.09** (0.17)	(0.03)	3.05	0.002	0.0304	0.1404
Dangerous worldview on trust in authoritarian leader	0.11 (0.11)	(0.08)	1.48	0.140	–0.0380	0.2673	0.06 (0.05)	(0.06)	0.90	0.371	–0.0668	0.1788
Authoritarianism on trust in authoritarian leader	**0.68** (0.32)	(0.16)	4.15	<0.001	0.3557	1.0023	**0.46** (0.23)	(0.11)	4.06	<0.001	0.2081	0.6447
Age on dangerous worldview	−0.01 (−0.09)	(0.01)	–1.19	0.236	–0.0198	0.0049	−**0.01** (−0.14)	(0.00)	–3.05	0.003	–0.0178	–0.0038
Age on authoritarianism	0.00 (0.04)	(0.00)	0.60	0.551	–0.0040	0.0075	**0.00** (0.10)	(0.00)	2.21	0.028	0.0005	0.0082
Age on trust in authoritarian leader	−0.01 (−0.12)	(0.01)	–1.67	0.097	–0.0232	0.0019	−0.01 (−0.09)	(0.00)	–1.75	0.081	–0.0163	0.0009
Sex on dangerous worldview	−0.21 (−0.11)	(0.14)	–1.46	0.1448	–0.4868	0.0720	−**0.20** (−0.10)	(0.09)	–2.18	0.030	–0.3733	–0.0195
Sex on authoritarianism	**0.19** (0.19)	(0.07)	2.92	0.004	0.0625	0.3234	**0.17** (0.15)	(0.05)	3.47	0.001	0.0743	0.2691
Sex on trust in authoritarian leader	0.01 (0.01)	(0.15)	0.09	0.926	–0.2774	0.3048	**0.43** (0.19)	(0.11)	4.06	<0.001	0.2369	0.6810
Political orientation on dangerous worldview	**0.09** (0.15)	(0.04)	2.00	0.047	0.0013	0.1733	**0.06** (0.10)	(0.03)	2.25	0.025	0.0081	0.1180
Political orientation on authoritarianism	**0.11** (0.37)	(0.02)	5.44	<0.001	0.0709	0.1516	**0.14** (0.41)	(0.02)	9.17	<0.001	0.1109	0.1715
Political orientation on trust in authoritarian leader	**0.12** (0.20)	(0.05)	2.68	0.008	0.0327	0.2166	0.04 (0.06)	(0.04)	1.06	0.289	–0.0339	0.1137
Political interest on dangerous worldview	–	–	−−	−−	−−	−−	−**0.20** (−0.18)	(0.05)	–3.70	<0.001	–0.3067	–0.0939
Political interest on authoritarianism	–	–	−−	−−	−−	−−	−**0.08** (−0.13)	(0.03)	–2.79	0.006	–0.1434	–0.0249
Political interest on trust in authoritarian leader	–	–	−−	−−	−−	−−	−**0.12** (−0.09)	(0.07)	–1.82	0.070	–0.2543	0.0099
**Indirect effects**												
Economic crisis on trust in authoritarian leader via dangerous worldview	0.03	(0.02)			–0.0068	0.0748	0.01	(0.02)			–0.0181	0.0492
Economic crisis on trust in authoritarian leader via authoritarianism	−0.06	(0.03)			–0.1160	0.0000	−0.02	(0.01)			–0.0536	0.0028
Economic crisis on trust in authoritarian leader via dangerous worldview and authoritarianism	**0.01**	(0.01)			0.0022	0.0328	**0.01**	(0.00)			0.0025	0.0218
SES on trust in authoritarian leader via dangerous worldview	−0.03	(0.02)			–0.0829	0.0076	−0.02	(0.02)			–0.0635	0.0248
SES on trust in authoritarian leader via authoritarianism	−**0.07**	(0.04)			–0.1494	–0.0075	−0.03	(0.02)			–0.0666	0.0018
SES on trust in authoritarian leader via dangerous worldview and authoritarianism	−**0.02**	(0.01)			–0.0391	–0.0013	−**0.01**	(0.01)			–0.0279	–0.0034
**Total effects**												
Economic crisis on trust in authoritarian leader	−0.10	(0.09)	–1.06	0.292	–0.2866	0.0868	0.05	(0.06)	0.84	0.403	–0.0710	0.1764
SES on trust in authoritarian leader	−**0.34**	(0.12)	–2.92	0.004	–0.5771	–0.1118	−0.04	(0.08)	–0.52	0.603	–0.2063	0.1200

**Unstandardized coefficients presented (standardized coefficients in brackets). Significant coefficients emphasized in bold. LL, lower limit; UL, upper limit; CI, confidence interval. Sex: 1 = woman, 2 = man*.*

### Discussion

As hypothesized, the lower participant SES or the more threatened participants felt as a consequence of the economic crisis, the more they tended to see the world as a dangerous and unpredictable place; this worldview was associated with a more authoritarian ideology, which in turn was linked to greater trust in leaders with an authoritarian style. Hence, our data supported and broadened the approach of the dual-process model (Duckitt, 2001), showing that economically threatening contexts (i.e., the economic crisis and individuals’ SES) have an effect on how we see the world around us and that this view is related to support for authoritarian ideologies and leaders. Our results also revealed that the threat from the economic crisis and belonging to a low social class had similar psychological consequences. This may be because both situations entail economic distress and greater insecurity for individuals, as suggested by the higher dangerous worldview derived from both situations. Yet, there are also important differences between both economic contexts. The main one is probably that SES is a more stable and long-lasting condition in the life of individuals (Moya and Fiske, 2017) that implies having less access to all kinds of resources; by contrast, the crisis is a temporary condition that can affect all social classes. However, we consider that, despite the differences between both contexts, there may be other variables related to the perceived social environment that are common to both contexts and have a similar effect on adherence to authoritarianism. In our research we hypothesized that this variable would be perceived control of the social and political environment and explored this variable in Study 2.

Finally, although the results supported our hypotheses, a limitation of the present study may be the type of measure used to record the perceived threat from the economic crisis (two items). It would be interesting to try to obtain the same results with a measure that encompasses the construct in a more comprehensive way.

## Study 2

Study 2 explored in greater detail the reasons why dangerous worldview derived from economically threatening contexts (i.e., the economic crisis and SES) promotes authoritarianism and trust in authoritarian leaders. As we just explained, we argued that low perceived control of the social and political context may be an antecedent of support for authoritarianism and authoritarian leaders as a means to regain this feeling of control (Hypothesis 3), threatened by the view of the world as an insecure and unpredictable place. Previous studies (Fritsche et al., 2017) have shown that economic threat (e.g., having low SES or changing to a lower social class as a consequence of the Great Recession of 2008) decreases perceived personal control, which promotes both positive responses to the crisis (e.g., collective action) and palliative and negative responses (e.g., greater ethnic prejudice). In Study 2, we explored whether dangerous worldview derived from economic threat (i.e., low SES and economic crisis) is associated with low perceived control of the sociopolitical context, and whether low sociopolitical control is related in turn to palliative responses aimed at regaining control, such as a more authoritarian ideology. This mediation was not pre-registered, even as an exploratory line of investigation. In this study we considered a more comprehensive measure of threat from the economic crisis. Thus, we expected to observe again an indirect effect of the economic crisis and participant SES on trust in authoritarian leaders through dangerous worldview and authoritarianism (Hypotheses 1 and 2), which would contribute to reinforce the findings and conclusions of Study 1.

### Materials and Methods

The method and Hypotheses 1–3 were preregistered in the Open Science Framework on July 18, 2018 and may be consulted in the following link: https://osf.io/pm8u4/?view_only=81c56cebeb2f43a4b7d4f3dae1907329.

This link also includes the Supplementary Materials, datasets, and syntax appertaining to Study 2. Additionally, in Study 2, we deviated from pre-registration to explore the relationship between dangerous worldview and authoritarianism through perceived sociopolitical control.

#### Participants

We calculated the necessary sample size prior to data collection. As regards the hypothesis on the correlation between perceived sociopolitical control and authoritarianism (Hypothesis 3), we established a minimum of 193 participants to test a two-tailed bivariate correlation, considering a moderate-low correlation coefficient between both variables (*r* = 0.20), a statistical power of 0.80 and a 95% confidence interval (G^∗^Power analysis; Faul et al., 2009). To calculate the sample size needed to test the indirect effect of the economic crisis and SES on trust in an authoritarian leader through dangerous worldview and authoritarianism (Hypotheses 1 and 2), we used the simulations of Monte Carlo software (Schoemann et al., 2017), considering the correlations between variables and standard deviations found in Study 1, a power of 0.80 and a 95% confidence interval. This analysis showed that at least 390 valid cases were needed, so we set this sample size in order to replicate the findings of Study 1 (Hypotheses 1 and 2), to verify the negative relationship between sociopolitical control and authoritarianism (Hypothesis 3), and to explore whether sociopolitical control mediates the relationship between dangerous worldview and authoritarianism. The sample was composed of 486 participants, of whom 413 (56.7% women and 43.3% men; *M*_age_ = 30.98, *SD*_age_ = 13.05) were considered for the analyses after applying the exclusion criteria (citizenship other than Spanish; age under 18 or over 70 years; not having answered the measure of trust in an authoritarian leader; and outliers derived from the analysis of atypical cases; see Supplementary Material and Supplementary Table 2 for further details concerning the exclusion procedure for participants). Educational level and income level were similar to those of participants in Study 1 (Table 1).

#### Procedure

In Study 2, which was also correlational, we administered a paper questionnaire to people from the general population at the bus station of a city in southern Spain. Data collection started on July 19, 2018 and ended on August 04, 2018. As in Study 1, all participants gave consent to participate voluntarily in the study, in accordance with the Declaration of Helsinki. Finally, participants were given the contact details of the person in charge of the study in case they wished to request more information on its objectives and results.

#### Measures

Participants completed the same scales used in Study 1 for the variables dangerous worldview (α = 0.76), authoritarianism (α = 0.74), trust in an authoritarian leader (α = 0.92), and SES. The questionnaire also included new scales to measure the threat from the economic crisis and perceived sociopolitical control. It included the usual covariates (i.e., age, sex, nationality, political orientation [from 1, *far left*, to 10, *far right*)]) and also a new measure to control for participants’ interest in politics (the details of this measure can be found in the Supplementary Material).

##### Threat from the economic crisis

We used the Financial Threat Scale (Marjanovic et al., 2013), adapted to Spanish by us. This measure allowed us to approach the threat from the economic crisis in a more comprehensive way. Specifically, participants were asked to think about their current financial situation and report to what extent (a) they felt uncertain, (b) they felt at risk, (c) they felt threatened, (d) they thought about it and (e) they were concerned about the situation, with a single overall score (α = 0.86). In our study we adapted the instructions so that participants would answer thinking about their financial situation as a consequence of the economic crisis. The measure had a five-point Likert response format (from 1, *not at all*, to 5, *very much*), and higher scores reflected higher threat.

##### Perceived sociopolitical control

We used the Sociopolitical Control subscale of the Spheres of Control Scale (SOC; Paulhus, 1983), adapted to Spanish for the present research. Following the recommendations of Spittal et al. (2002), we used the Sociopolitical Control subscale of the first version of the SOC (Paulhus, 1983). It is composed of 10 items that assess to what extent individuals feel that citizens have an influence on their social environment and decisions taken at a political level (e.g., “The average citizen can have an influence on government decisions”; α = 0.61). It had a seven-point Likert response format (from 1, *totally disagree*, to 7, *completely agree*). We recoded items worded inversely so that higher scores reflected higher perceived sociopolitical control.

### Results

First, we analyzed the correlations between the variables of interest: threat from the economic crisis, participant SES, dangerous worldview, authoritarianism, perceived sociopolitical control, trust in an authoritarian leader and the covariates (Table 2). Scores on perceived sociopolitical control were negatively correlated with scores on authoritarianism, which confirmed Hypothesis 3. Sociopolitical control was also negatively correlated with dangerous worldview. Again, the correlation analysis supported the relationships expected according to Hypotheses 1 and 2: the new measure of threat from the economic crisis positively correlated with dangerous worldview, while the correlation between participant SES and dangerous worldview was negative; dangerous worldview also positively correlated with authoritarian ideology, and both were correlated with higher trust in an authoritarian leader.

#### Indirect Effects of Threat From the Economic Crisis and SES on Trust in an Authoritarian Leader Through Dangerous Worldview and Authoritarianism

**FIGURE 3:**
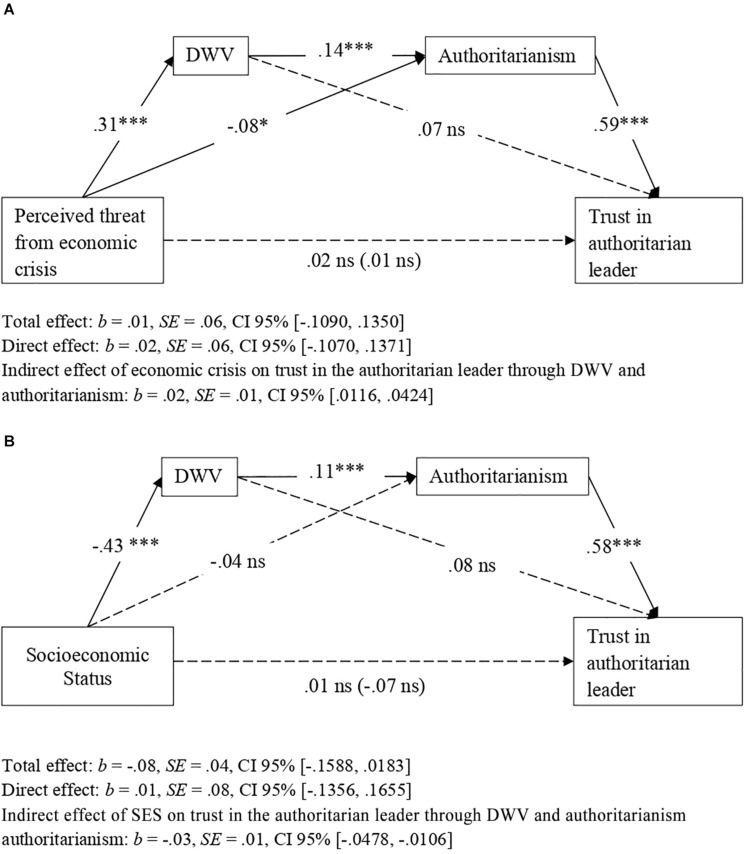
Indirect effects of perceived threat form economic crisis **(A)** and SES **(B)** on trust in an authoritarian political leader via dangerous worldview (DWV) and authoritarianism (Study 2). Figure shows the effects without consideration of covariates. Non-significant effects appear with dashed lines. Unstandardized coefficients presented. *p < 0.05, ***p < 0.001.

To replicate the indirect effects of threat from the economic crisis and participant SES on trust in an authoritarian leader through dangerous worldview and authoritarianism (Hypotheses 1 and 2), we conducted two serial mediation analyses with PROCESS software (Model 6, bootstrapping procedure, 10,000 repeats, 95% CI): in one analysis we considered perceived threat from the economic crisis as a predictor variable, and in the other we included participant SES as a predictor variable. Results confirmed Hypotheses 1 and 2 (Figure 3). In fact, both threat from the economic crisis and low SES were associated with higher dangerous worldview, which in turn was associated with higher scores on authoritarianism; finally, higher authoritarianism was related to greater trust in an authoritarian leader. Interestingly, the total effects of threat from the economic crisis and participant SES on trust in an authoritarian leader were not significant. In other words, the relationship between, on the one hand, threat from the economic crisis and participant SES, and, on the other, trust in an authoritarian leader, was entirely accounted for by dangerous worldview and authoritarian ideology (Figure 3). Further, the indirect effect of both threat from the economic crisis and participant SES on trust in an authoritarian leader, attributable to dangerous worldview and authoritarianism, remained statistically significant when the covariates and both predictors were included in the analysis (Table 3). All the paths of the indirect effect were statistically significant, both for the effect of the economic crisis and the effect of SES, therefore meeting the requirements established by Yzerbyt et al. (2018) to correctly identify indirect effects (see Figure 3 and Table 3).

#### Relationship Between Dangerous Worldview, Sociopolitical Control, and Authoritarianism

**FIGURE 4:**
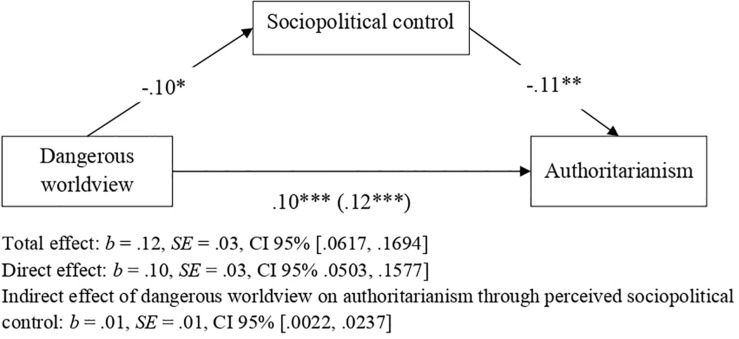
Direct and indirect effect of dangerous worldview on authoritarianism through perceived sociopolitical control. Unstandardized coefficients presented. *p < 0.05, **p < 0.01, ***p < 0.001.

**FIGURE 5:**
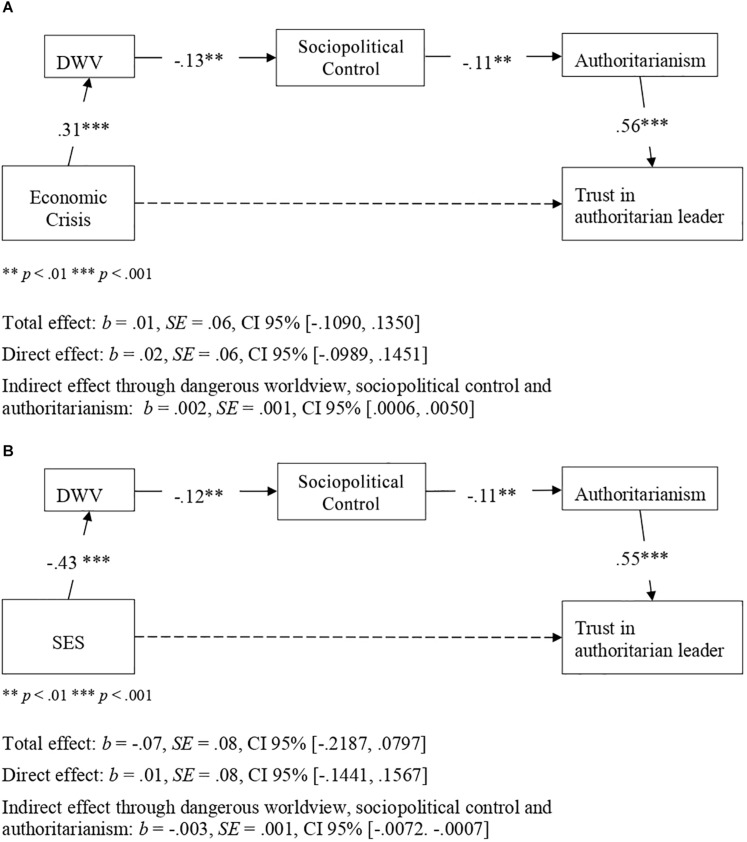
Indirect effect of economic crisis **(A)** and participant’ SES **(B)** on trust in authoritarian leader through dangerous worldview, perceived sociopolitical control and authoritarianism. Unstandardized coefficients presented.

To explore whether dangerous worldview was associated with greater authoritarianism through lower perceived sociopolitical control, we conducted a mediation analysis with PROCESS software (Model 4, bootstrapping procedure, 10,000 repeats, 95% CI), considering dangerous worldview as a predictor variable, perceived sociopolitical control as a mediating variable and authoritarianism as a criterion variable. As shown in Figure 4, the higher the level of dangerous worldview, the greater is the level of authoritarian ideology and the lower is that of perceived sociopolitical control; in turn, lower perceived control was associated with a more authoritarian ideology. In addition, the indirect effect of dangerous worldview on authoritarianism through perceived sociopolitical control was statistically significant, showing that perceived sociopolitical control partially mediated the relationship between dangerous worldview and authoritarianism. We also explored the full model for the indirect effect of economic crisis and participant SES on trust in an authoritarian political leader, considering dangerous worldview, perceived sociopolitical control, and authoritarianism as mediating variables. In this way, we intend to explore the possibility of integrating the approaches of the two models on which this research is based: the dual-process model (Duckitt. 2001) and the compensatory control model (Kay et al., 2008). As Figure 5 shows, there was statistical significance in the indirect effect of both economic crisis (perceived threat) and participant SES on trust in an authoritarian political leader through dangerous worldview, perceived sociopolitical control, and authoritarianism. In addition, all paths were statistically significant, according to Yzerbyt et al. (2018).

We repeated these analyses with the inclusion of both predictors, together, in addition to the covariates, in order to check whether the full model’s indirect effect remained significant. Results are shown in Table 4. All paths remained statistically significant, with the exception of the effect of sociopolitical control on authoritarianism. Specifically, sociopolitical control had no effect on authoritarianism when political orientation was included (see Supplementary Table 4 for further details). Accordingly, the indirect effect of both economic crisis and SES was no longer significant after the inclusion of political orientation.

**TABLE 4:** Full model for the direct, indirect and total effect of threat by economic crisis and SES on trust in an authoritarian political leader including both predictors in the analysis and controlling by age, sex, political orientation and political interest.

	*B* (β)	(*SE*)	*t*	*p*-value	LL 95% CI	UL 95% CI
**Direct effects**						
Economic crisis on dangerous worldview	**0.26** (0.24)	(0.05)	5.08	<0.001	0.1585	0.3589
Economic crisis on sociopolitical control	0.02 (0.02)	(0.05)	0.43	0.668	–0.0705	0.1099
Economic crisis on authoritarianism	−0.05 (−0.08)	(0.03)	–1.68	0.094	–0.1051	0.0082
Economic crisis on trust in authoritarian leader	0.05 (0.04)	(0.06)	0.82	0.414	–0.0732	0.1775
SES on dangerous worldview	−**0.38** (−0.28)	(0.07)	–5.73	<0.001	–0.5174	–0.2530
SES on sociopolitical control	−**0.12** (−0.11)	(0.06)	–1.98	0.049	–0.2407	–0.0008
SES on authoritarianism	−**0.08** (−0.10)	(0.04)	–1.98	0.048	–0.1521	–0.0006
SES on trust in authoritarian leader	0.01 (0.01)	(0.09)	0.16	0.870	–0.1539	0.1819
Dangerous worldview on sociopolitical control	−**0.10** (−0.12)	(0.04)	–2.24	0.026	–0.1870	–0.0120
Dangerous worldview on authoritarianism	**0.08** (0.15)	(0.03)	2.92	0.004	0.0269	0.1376
Dangerous worldview on trust in authoritarian leader	0.05 (0.04)	(0.06)	0.74	0.458	–0.0767	0.1699
Sociopolitical control on authoritarianism	−0.03 (−0.05)	(0.03)	–0.99	0.322	–0.0951	0.0313
Sociopolitical control on trust in authoritarian leader	−0.10 (−0.07)	(0.07)	–1.42	0.156	–0.2404	0.0385
Authoritarianism on trust in authoritarian leader	**0.45** (0.22)	(0.11)	3.99	<0.001	0.2288	0.6728
**Covariates**						
Age on dangerous worldview	−**0.01** (−0.14)	(0.00)	–3.05	0.003	–0.0178	–0.0038
Age on sociopolitical control	−**0.01** (−0.12)	(0.00)	–2.33	0.020	–0.0135	–0.0011
Age on authoritarianism	**0.00** (0.10)	(0.00)	2.07	0.039	0.0002	0.0080
Age on trust in authoritarian leader	−0.01 (0.10)	(0.00)	–1.90	0.058	–0.0170	0.0003
Sex on dangerous worldview	−**0.20** (−0.10)	(0.09)	–2.18	0.030	–0.3733	–0.0195
Sex on sociopolitical control	−0.09 (−0.06)	(0.08)	–1.12	0.261	–0.2437	0.0664
Sex on authoritarianism	**0.17** (0.15)	(0.05)	3.40	0.001	0.0713	0.2664
Sex on trust in authoritarian leader	**0.42** (0.19)	(0.11)	3.77	<0.001	0.2006	0.6371
Political orientation on dangerous worldview	**0.06** (0.10)	(0.03)	2.25	0.025	0.0081	0.1180
Political orientation on sociopolitical control	−**0.08** (−0.17)	(0.02)	–3.35	0.001	–0.1303	–0.0339
Political orientation on authoritarianism	**0.14** (0.41)	(0.02)	8.87	<0.001	0.1078	0.1693
Political orientation on trust in authoritarian leader	0.03 (0.05)	(0.04)	0.87	0.387	–0.0416	0.1071
Political interest on dangerous worldview	−**0.20** (−0.18)	(0.05)	–3.70	<0.001	–0.3067	–0.0939
Political interest on sociopolitical control	**0.22** (0.24)	(0.05)	4.61	<0.001	0.1269	0.3156
Political interest on authoritarianism	−**0.08** (−0.12)	(0.03)	–2.49	0.013	–0.1380	–0.0162
Political interest on trust in authoritarian leader	−0.10 (−0.08)	(0.07)	–1.46	0.145	–0.2359	0.0347
**Indirect effects**						
Economic crisis on trust in authoritarian leader via dangerous worldview, sociopolitical control and authoritarianism	0.00	(0.00)			–0.0004	0.0014
SES on trust in authoritarian leader via dangerous worldview, sociopolitical control and authoritarianism	−0.00	(0.00)			–0.0021	0.0005
**Total effects**						
Economic crisis on trust in authoritarian leader	0.05	(0.06)	0.84	0.403	–0.0710	0.1764
SES on trust in authoritarian leader	−0.04	(0.08)	–0.52	0.603	–0.2063	0.1200

**Unstandardized coefficients presented (standardized coefficients in brackets). Significant coefficients emphasized in bold. LL, lower limit; UL, upper limit; CI, confidence Interval. Sex: 1 = woman, 2 = man*.*

### Discussion

In Study 2, we replicated the results obtained in Study 1: perceived threat from the economic crisis was associated with a dangerous worldview, which in turn was related to higher authoritarianism, and finally to a greater trust in an authoritarian leader (Hypothesis 1). This result was corroborated with a more comprehensive measure of perceived threat from the economic crisis, thus providing greater methodological guarantees. Participants with low socioeconomic status also perceived the world as a dangerous place, with the same consequences on authoritarianism and trust in an authoritarian leader described regarding the economic crisis (Hypothesis 2). In contrast with Study 1, participant SES did not have a total effect on trust in an authoritarian leader in Study 2. Instead, as happened with the economic crisis, we found an indirect pathway (through dangerous worldview and authoritarianism) between participant SES and trust in an authoritarian leader. This difference between both studies may results from other contextual factors such as individuals’ trust in politics at the two different moments when Studies 1 and 2 were conducted. In fact, according to data of the Spanish Center for Sociological Research (CIS; n.d.), both trust in politics and the general assessment of the political situation in Spain were more negative when Study 1 was conducted (March 2017) than when Study 2 was performed (July 2018). In addition to dangerous worldview and authoritarianism, lower trust in politics may have led participants with low socioeconomic status to consider a leader with an authoritarian style as the best option for Spain to overcome the problems derived from the economic crisis in Study 1.

The results of Study 2 also reveal that the perception of the social world as insecure and unpredictable—due to the economic crisis or due to the belonging to a disadvantaged social status—is related to the feeling that individuals cannot influence their social and political context (i.e., lower control), which is, in turn, associated with an increase in authoritarianism as a palliative strategy to regain control. These results are similar to those of previous studies that have found that the economic threat decreases perceived personal control, which in turn promotes palliative responses to the crisis such as prejudice (Fritsche et al., 2017). These results also support the approaches of the compensatory control model (Kay et al., 2008). As the model postulates, when individuals perceive that their control is under threat, they may seek external control sources such as authoritarianism and ultimately trust in authoritarian leaders. Moreover, our results expand on previous findings, since they reveal that this compensatory mechanism functions with a different type of perceived control: sociopolitical control. However, the link between sociopolitical control and authoritarianism disappears when controlling for participants’ political orientation. The items of the sociopolitical control scale measure the belief that citizens, in a collective way, can influence their sociopolitical context. Two main ideas are implicit in these items: (a) citizens’ ability to organize collectively and (b) the intention to promote changes in society and to challenge authority. Conservative people are characterized by spurning social change and by conforming to authorities. Literature consistently shows that right-wing people tend to engage less in social protests comparing with left-wing people (Marsh, 1977; Barnes et al., 1979; Klingemann, 1979; Bernhagen and Marsh, 2007; van der Meer et al., 2009; Torcal et al., 2016). Thus, it is possible that right-wing participants might score lower in perceived sociopolitical control than left-wing participants. Furthermore, according to the compensatory control model, low sense of control results in conservative ideologies because these promote social order and stability. Previous findings also show that conservative people, when compared with liberal people, score higher in need for closure. Thus, the literature suggests that, at baseline, right-wing people feel less control over their environment than left-wing people. The above considered, perceived sociopolitical control and political orientation may to some degree overlap in our research.

## General Discussion

The rise and consolidation of political leaders with a marked authoritarian style is a concerning change in the social and political scenario over the last few years. The results obtained in our studies empirically demonstrate that the economic crisis and socioeconomic status (SES), two macrosocial variables, can promote trust in authoritarian political leaders through various psychological processes.

Previous studies have shown a positive relationship between perceived social threat and authoritarianism (e.g., Duckitt and Fisher, 2003; Mirisola et al., 2014) and also between such perceived threat and the preference for authoritarian far-right political parties (e.g., Merolla and Zechmeister, 2009; Aichholzer and Zandonella, 2016) and/or authoritarian leaders (e.g., Laustsen and Petersen, 2017). Yet, most of these studies have analyzed the social threat mainly related to aspects such as immigration, crime rates, armed conflict situations or terrorist attacks.

Our research shows that perceived social threat from the economic crisis—understood as economic insecurity and difficulties and perceived uncertainty about the future—is also associated with authoritarianism and trust in authoritarian leaders, defined in neutral terms regarding the left–right- political axis. The results obtained in Studies 1 and 2 also show that perceived threat from the economic crisis was associated with a higher view of the world as a dangerous, insecure and unpredictable place. This view was in turn associated with a more authoritarian ideology, which finally was associated with greater trust in an authoritarian leader described to solve the problems related to the economic crisis in Spain. These results therefore provide empirical support to the dual-process model with a specific type of threat (i.e., the consequences of the economic crisis) and with trust in an authoritarian leader—with unspecified political orientation—instead of support for conservative policies as a criterion variable, as is usually considered in studies in the framework of the dual-process model.

Our research also provides further insight on the similarities between the two macrosocial variables considered: experiencing a major impact of the economic crisis and belonging to a low social class with few possibilities to access material, educational or other resources. Results show that both economically threatening contexts were directly associated with dangerous worldview. Moreover, this perception of the social environment seems to be the key mechanism for understanding why two types of economic threat, one relatively stable (i.e., socioeconomic status) and one that is temporary and can affect any social class (i.e., the economic crisis) share other similar consequences such as adherence to authoritarianism and trust in strong and dominant leaders. It is likely that the common element in both contexts that favors dangerous worldview is uncertainty and insecurity in the face of job loss or worsening working conditions. In other words, dangerous worldview would be motivated by a feeling of insecurity that is generated either by being unemployed or in a precarious work situation, or by the possibility of being in such a situation. Future studies might investigate this question: specifically, analyzing the relationship between (fear of) unemployment and dangerous worldview.

Our results provide greater insight on the mechanisms through which (low) social class has been found to be related to conservative ideology in various studies (Jost and Hunyady, 2005; Napier and Jost, 2008; Carvacho et al., 2013) and also to support for authoritarian parties, both right-wing (Corbetta et al., 2018; Tuticì and von Hermanni, 2018) and left-wing (Corbetta et al., 2018). Unlike these prior studies, our research shows that the simple relationship (total and direct effects) between belonging to a disadvantaged socioeconomic status—understood in our research as lower income and lower level of education—and authoritarianism is rather weak and/or not significant. However, we consistently found an indirect effect between both variables. Thus, our results are not contrary to those previously found (e.g., Napier and Jost, 2008; Carvacho et al., 2013), but rather show that the relationship between SES and authoritarianism occurs through the perception of the world as a dangerous place. In fact, some of these authors, from a motivated social-cognitive perspective, propose that working-class people can embrace conservative ideologies as a coping strategy for reducing the dissonance, uncertainty, or insecurity derived from their socioeconomic condition (Jost et al., 2003). Our results also point in this direction, suggesting that low-SES people may develop authoritarian attitudes as a coping and adaptation strategy in a world perceived as dangerous. Based on extensive social cognition literature, we may understand dangerous worldview as a mental representation (Smith, 1998) of our social world, which is elaborated and consolidated from our experiences and interactions with others in a given context. Differences in material resources and in social and cultural capital between high- vs. low-SES people can influence the mental representation of the world developed by people belonging to each group (Manstead, 2018). In turn, this mental representation of the world may act as a frame of reference for interpreting events that occur on social and global levels, thereby influencing people’s sociopolitical attitudes and behaviors (Kunda, 2002). Our results suggest, therefore, that it is neither scarcity of resources nor low educational level *per se* that is related to authoritarianism, but the negative mental representation of the world that is promoted by these socioeconomic conditions.

A second mechanism that helps understand the relationship between threat from the economic crisis and social class on one side and authoritarianism and trust in authoritarian political leaders on the other is perceived control. In Study 2 we found the greater the feeling that the world is an insecure and unpredictable place, the lower the feeling that citizens can do something to change the situation. This low perceived sociopolitical control makes people likely to support ideologies that emphasize the need to subject themselves to a leader that makes decisions for them and to justify aggression toward people who pose a threat (i.e., authoritarianism); this is likely to increase trust in political leaders with an authoritarian style that promote this type of beliefs. The present research thus provides empirical support to the assumptions of the compensatory control model using a different dimension of perceived control (i.e., sociopolitical control) from that considered by prior research. The results obtained are also consistent with the idea that individuals seek external control sources such as the government (or, in our case, an authoritarian leader) when they cannot regain the feeling of control through the social self (Fritsche et al., 2013). In Study 2, the lower participants’ perception that citizens have the ability to influence sociopolitical issues, the higher their authoritarianism and, consequently, their trust in an authoritarian leader. This low perceived sociopolitical control may reflect participants’ belief that citizens are not an agentic group that they can identify with (i.e., social self) to regain their feeling of control. This is likely to lead them to resort to authoritarian ideologies and leaders as an external source of control. It would be interesting to include a measure of personal control in future studies to verify if dangerous worldview as a consequence of the economic crisis threatens both personal and sociopolitical control, leading to adherence to authoritarian ideologies and leaders as a compensatory control mechanism. Interestingly, participants’ political orientation nulled the relationship between sociopolitical perceived control and authoritarianism (while it does not affect the link between dangerous worldview and authoritarianism). As mentioned above, this may result from an overlap between the two variables, as conservative ideology is conceptually linked to low sense of control and low engagement in social change. There is a further possibility: despite the baseline in perceived control remaining lower among right-wing participants, left-wing participants are also able to experience decreases in this variable. As the “rigidity of the right” hypothesis (Tetlock, 1984) suggests, the need for structure and order (i.e., need for closure or, in our case, need for control) may bring both right- and left-wing people closer to more conservative attitudes and ideologies (e.g., Jost et al., 2003). In future studies, it might be interesting to measure perceived sociopolitical control in supporters of both left and right political parties before and after manipulating this variable. In this way, it may be observable whether it is in fact the change in perceived sociopolitical control, rather than the absolute level of control, that is related to authoritarianism among people of various political orientations.

Taken together, our results are similar to those of other studies that attribute the emergence of authoritarianism to individuals’ perceptions of anomie. From a psychosocial approach, Teymoori et al. (2017) define anomie as the perception that society is breaking down (similar to dangerous worldview). Perception of anomie may be presumed to prevent people from satisfying their need for control, thus reducing their psychological well-being (contraction of personal self) and their connection and involvement with society (contraction of social self). Contraction of personal self leads people to reliance on control-restoring ideologies, such as authoritarianism; while contraction of social self encourages people to seek to belong to smaller groups that provide them with strong, safe, and secure ties (e.g., emergent politicized groups) (Teymoori et al., 2017). In our studies, low sociopolitical control can be understood as contraction of both personal and social self. Therefore, our research provides empirical support for the psychological analysis of anomia and their association with authoritarianism (Teymoori et al., 2017).

Another important aspect of our research is that we analyzed both authoritarian ideology and trust in an authoritarian leader without referring to the left–right political axis. This strategy has allowed us to find that the effect of an economically threatening context (i.e., the crisis and SES) on trust in authoritarian leaders through dangerous worldview persists even when political orientation of participants is controlled for. We consider this to be a significant contribution to the study of authoritarianism and the separation of the latter from political orientation. Our results also support the conclusions of the authors of the ASC scale that Aggression (followed by Submission) is the most consistent component of authoritarianism and perhaps the key factor to assess this construct regardless of the political orientation of participants. This aspect may be of interest in future studies aimed at comparing trust in left- and right-wing authoritarian leaders. Aggression and submission are the most significant topics in most theories on authoritarianism. Though the two dimensions seem to conflict in theoretical terms, aggression and submission can coexist because they are directed at different targets: aggression is oriented toward outgroups while submission is oriented toward the ingroup and/or the leader. Thus, our results are in accordance with situational approaches to authoritarianism (e.g., Oesterreich, 2005), as they suggest that individuals (whatever their political orientation) react to a threatening context by submitting to authorities and by showing hostility toward those that threaten authorities or social order.

Finally, in this research we tried to integrate the approaches of two different models that explain the relationship between threat and conservative ideology (i.e., the dual-process model, Duckitt, 2001; and the compensatory control mechanism, Kay et al., 2008), applying them to the study of the relationship between economic crisis/SES and trust in authoritarian political leaders, both right- and left-wing. Overall, our results suggest that it is possible to integrate both proposals and that low perceived sociopolitical control may be involved in the relationship between dangerous worldview and authoritarianism. On the one hand, as postulated by the dual-process model, it could be understood that the combination of certain personality characteristics and socialization in threatening environments leads individuals to develop a view of the world as a dangerous place, which would imply a relatively chronic lack of perceived control among them and is likely to lead to adherence to authoritarianism as a compensatory control mechanism. On the other hand, in line with the compensatory control model, exposure to an unpredictable and intense threatening context such as the economic crisis is likely to modify individuals’ perception of their environment leading them to view it as more dangerous, which would imply lower perceived control and consequently the search for authority as a way of reestablishing it.

### Practical Implications

The results of this research provide valuable insight for the applied context. Given that negative socioeconomic conditions predict a higher dangerous worldview, ensuring that the population have better access to all types of resources can avoid the development of perceptions of the social environment as being insecure, threatening and unpredictable. This can be used as a protective shield to prevent the adherence to authoritarian ideologies and leaders. Even when socioeconomic conditions are good, people can develop the perception of the world as a dangerous place, especially because of the influence of certain media outlets, political speeches or fake news, leading to the risk of strengthening authoritarian ideologies and leaders (e.g., Mols and Jetten, 2016). It is important to highlight that the results obtained may be particularly relevant in the context of the current health crisis caused by the spread of Covid-19 and the harsh economic crisis that the pandemic has enhanced. The Covid-19 crisis has undoubtedly influenced our perception that the world is not as safe and predictable as we thought, which is compounded by the huge campaign of disinformation and fake news that circulate through social media promoting insecurity, uncertainty and fear.

Our results also suggest that the development of actions aimed at increasing the control of citizens over their social and political context can reduce support for authoritarian ideologies and leaders as a compensatory mechanism. This applies even to citizens with left-wing political orientation, preventing them from embracing authoritarian ideologies or identifying with authoritarian parties and leaders that promote conservative political measures that they would not agree with *a priori*.

### Limitations and Future Research

Although our research provides interesting and new results on the rise of authoritarianism in our society, it also has limitations.

As regards our dependent variable, we only described one authoritarian leader. Therefore, we were not able to determine whether participants who feel a greater threat from the economic crisis or who belong to a low social class trust an authoritarian leader more than a democratic one. Future studies should compare both styles of political leadership to draw conclusions on the reasons why individuals trust authoritarian versus democratic leaders. Moreover, although our results show the indirect effect of threat from the economic crisis and social class on trust in a leader, overall, the scores on this measure were noticeably low (see Table 2). The low trust in the leader that was exhibited by participants may be due to several reasons. On the one hand, it is likely that participants in our research, who were from the general population, simply reflected citizens’ low trust in their political leaders. On the other hand, the description of the leader may have given the impression that the leader was not interested in improving the population’s situation but, rather, motivated by self-interest (see the Supplementary Material). In future research, more work should be done on the description of the leader, in order to increase the leader’s trustworthiness and thereby endeavor to capture a greater variability of responses. Finally, the high percentage of people with a university degree may have influenced the scores obtained in the measure of trust in an authoritarian leader, reflecting a greater rejection of this type of political leadership by the more educated population.

A further limitation relates to the scale for perceived sociopolitical control, as used in Study 2. The reliability of this scale, although acceptable, was low. Thus, the relationships between dangerous worldview, perceived control, and authoritarianism that were found in our research should be replicated in future studies using a more appropriate measure of perceived sociopolitical control.

On the other hand, our research focuses on the link between economic threat, dangerous worldview, and authoritarianism. However, the dual process model sets out that competitive contexts favor competitive worldview which in turn leads to higher conservative ideologies and attitudes (Duckitt, 2001). Future studies should explore whether economic crisis and low SES are also associated with competitive worldview (owing to scarcity of resources and employment).

Finally, given the correlational nature of the two studies conducted, it is not possible to draw conclusions on the causal effect of perceived threat from the economic crisis on trust in authoritarian leaders. It should be noted that it is very difficult to conduct experimental studies in this field and that most research is correlational, although there are some longitudinal studies (Sibley and Duckitt, 2013) that concur with our findings. Nevertheless, experimental research is possible, for instance, through asking the participants to imagine that they have more or less resources, or that they are affected by the economic crisis. For example, Jetten et al. (2017) have shown how this kind of experimental design can be effective in manipulating socioeconomic status and economic instability. In addition, it would be possible for quasi-experimental studies to compare groups of individuals with high and low SES with respect to their reactions to experimentally manipulated economic threat.

As regards the implications for future research, apart from including leaders with a democratic style or the improvement of the dependent variable, our results suggest a few interesting lines of research. Studies 1 and 2 showed a negative relationship between socioeconomic status and trust in authoritarian leaders. Yet, other studies have found that both experiencing relative deprivation (which is more likely among people with low status) and experiencing relative gratification (which is more frequent among people with high status) are related with higher support for anti-migratory policies (this is known as the *v-curve*). Research has also revealed that fear of a potential future deprivation explains the relationship between relative gratification and support for such policies (Jetten et al., 2015). Therefore, it would be interesting to explore whether, in people with high SES, the threat implied by the consequences that the economic crisis may have in the future—rather than the currently perceived threat—is what triggers the dangerous worldview and authoritarianism. In respect of perceived control, it might prove interesting to undertake a deeper investigation of the link between dangerous worldview and perceived sociopolitical control, differentiating in regard to whether people do not exercise control over the social environment because they do not dare to or because they do not feel capable of doing so. Not daring to exercise control over the sociopolitical context could be related to a paternalistic and infantilized vision of citizenship, which would connect with the need for a protective figure (authoritarian leader) to take charge of the situation. Furthermore, the difference between “not be able to” and “not dare to” may relate to the effect of political orientation on the association between perceived control and authoritarianism. Are right-wing people more prone to not daring to challenge the social order? By contrast, when people on the left avoid challenging the social order, is it because they feel incapable of doing it? In a similar vein, political efficacy has been associated with political participation (Krauss, 2015). Thus, our results motivate the development of new studies on whether perceived control over the social and political context makes it possible to differentiate between different responses to the economic crisis situation: people who take on an active and participatory role aimed at bringing social change (i.e., people with high perceived sociopolitical control), and people who adopt a passive role and seek authoritarian leaders who take the helm of society and eliminate uncertainty (i.e., people with low perceived sociopolitical control).

In short, our research highlights that socioeconomic conditions influence the image we develop of the world in which we live. If the image is negative, it can lead people to support ideologies that can apparently make them change it, such as authoritarianism, which tends to convey clear-cut and oversimplified views of reality. This negative view of the social world can also lead individuals to adopt a more authoritarian ideology because it erodes the belief that the world is predictable and reduces people’s perceived control over their environment. Finally, the danger of supporting this type of ideologies is that it pushes people to trust strong, directive and even authoritarian political leaders, which can threaten the democratic system itself.

## Data Availability Statement

The raw data supporting the conclusions of this article can be found online at: https://osf.io/pm8u4/?view_only=81c56cebeb2f43a4b7d4f3dae1907329

## Ethics Statement

The two studies in this research were reviewed and approved by the Ethics Committee in Human Research (CEIH) of the University of Granada and carried out in compliance with the Ethical Standards of the 1964 Declaration of Helsinki.

## Author Contributions

LT-V, JR, and MM contributed to conception and design of the study and wrote sections of the manuscript. LT-V organized the database, performed the statistical analysis, and wrote the first draft of the manuscript. All authors contributed to manuscript revision, read, and approved the submitted version.

## Conflict of Interest

The authors declare that the research was conducted in the absence of any commercial or financial relationships that could be construed as a potential conflict of interest.

## Footnotes

1 Supplementary Material includes alternative analyses appertaining to (a) the interaction effect between economic crisis and SES on dangerous worldview, and (b) the indirect effect of dangerous worldview on trust in an authoritarian leader through the two components of authoritarianism.
