# Supplementary material for: Dangerous Worldview and Perceived Sociopolitical Control: Two Mechanisms to Understand Trust in Authoritarian Political Leaders in Economically Threatening Contexts
Source: Front Psychol. 2021 Mar 25;12:603116. doi: 10.3389/fpsyg.2021.603116 (PMC8027088; doi:10.3389/fpsyg.2021.603116)
Supplement: Supplementary file 1 [file Data_Sheet_1.docx]

Supplementary Material

This file include additional information (e.g., measures, description of the dependent variable, additional analysis, discussion of supplementary analysis) that are not detailed in the main document to facilitate understanding and reduce the length of the main document.

# Details for participants’ exclusion criteria and procedure.

## Study 1

The questionnaire used in Study 1 was designed online. Participants accessed the questionnaire through a link. Once they accessed the questionnaire, they first read a brief description of the research and gave their consent to voluntarily participate in the research. The participants then progressed through the different scales used to measure the variables of interest and record their sociodemographic data. Finally, they were thanked and informed that they were going to be redirected to another independent survey where they had to indicate their contact information (for participants entering the iPad giveaway), or the contact information of the student who had provided the link (for relatives of psychology students).

We thought that it would be easy for people to enter the questionnaire and start answering the scales, but that it would also be likely that many of these people would leave the questionnaire in the middle. As it is an online questionnaire to which the person answers in private, without the presence of the investigator, it is likely that they will not feel committed to completing the questionnaire and it will be easier for them to leave the investigation at any time and for any reason. Thus, we included participants in Study 1 who completed at least 90% of the progress, to ensure that our sample was composed of participants who had advanced at least to the end of the scales. On the other hand, to avoid those participants who filled out the survey solely motivated by the reward (iPad raffle or reward for students), we included an attention item with which we intended to identify participants who randomly answered the survey simply to arrive until the end and get the reward. The item read "Please check option 5 this time." Thus, only the participants who answered 5 in this item were included in the final sample. In sum, and considering the above, we established 4 exclusion criteria:

1. Participants under 18 years of age, since they must have the consent and approval of a legal guardian, and because the research was aimed at the adult population.

2. Non-Spanish participants, since the research referred to the economic and social context in Spain.

3. Participants who did not complete at least 90% of the questionnaire.

4. Participants who failed to answer the attention item.

Supplementary Table 1 shows the number of participants who met each of the above criteria. Finally, we explored the presence of multivariate outliers in our sample following the Mahalanobis Distance approach (Rousseeuw & Van Zomeren, 1990). No multivariate outliers were identified in the sample.

**Supplementary Table 1.** Details about participants’ exclusion criteria in Study 1.

|  | Total cases | Missing values | Total Sample |
| --- | --- | --- | --- |
| Age |  | 82 | 310 |
| < 18 | 0 |  |  |
| > 18 | 228 |  |  |
| Nationality |  | 82 | 310 |
| Spanish | 219 |  |  |
| Non-Spanish | 9 |  |  |
| Progress |  |  |  |
| < 90% | 82 | 82 | 310 |
| > 90% | 228 |  |  |
| Attention item |  |  |  |
| Correct response | 202 | 82 | 310 |
| Incorrect response | 26 |  |  |

## Study 2

We used paper questionnaires in Study 2 and participants did not receive any rewards for participating in the study. Thus, this time we did not include the attention item. We maintained the exclusion criteria about nationality (only Spanish people), age (participants between 18 and 70 years old), and progress (we excluded participants who did not complete the final dependent variable—trust in the authoritarian leader) (See Supplementary Table 2). We also explore the presence of multivariate outliers in our final sample, following the Mahalanobis Distance approach (Rousseeuw & Van Zomeren, 1990). Two participants were identified as multivariate outliers, so they were removed from the analyses.

**Supplementary Table 2.** Details about participants exclusion in Study 2.

|  | Total cases | Missing values | Total Sample |
| --- | --- | --- | --- |
| Age |  | 3 | 486 |
| < 18 | 6 |  |  |
| > 70 | 1 |  |  |
| Nationality |  | 11 | 486 |
| Spanish | 461 |  |  |
| Non-Spanish | 14 |  |  |
| Progress |  |  |  |
| < 90% | 44 | 44 | 486 |
| > 90% | 442 |  |  |

# Supplementary Material

## Political Leader Description (dependent variable)

Original version in Spanish:

“Se trata de un/a dirigente que tiene claro los objetivos que quiere alcanzar, y los pasos que quiere seguir para ello. Suele tomar decisiones de acuerdo a su propio criterio, sin considerar las sugerencias u opiniones de su círculo más cercano. Tampoco suele tener en cuenta la oposición de los miembros del Congreso a la hora de tomar decisiones respecto a las leyes o medidas políticas que deben aprobarse, ni dedica demasiado tiempo a dialogar y negociar con otros partidos u organizaciones ya que no suele ceder ni cambiar de postura respecto a los temas que considera importantes. Todas las decisiones importantes pasan por él/ella, prefiere supervisar y dirigir el trabajo de sus subordinados/as y no delegar demasiadas responsabilidades en los demás. Este/a dirigente se asegura de que sus propios intereses siempre se cumplan.”

Translation in English:

“This political leader is clear about the objectives he/she wants to achieve, and the steps he/she should follow for it. He/she usually makes decisions according to his/her own criteria, without considering the suggestions or opinions of his/her closest circle. This political leader doesn´t usually take into account the opposition of the members of Congress when making decisions regarding the laws or political measures that must be approved. He/she doesn´t devote too much time to dialogue and negotiate with other parties or organizations since he/she does not usually yield or change position on the issues that he/she considers important. He/she supervise all important decisions, and he/she prefers to supervise and direct the work of his/her subordinates and not delegate too many responsibilities to others. This leader makes sure that his/her own interests are always fulfilled.”

## Supplementary Measures (Study 2)

**1.2.1 Political Interest**

We included a measure of political interest to control their effect on perceived sociopolitical control, because participants that are more interested in politics should be more well-informed, and this could favor their sense of control about the social context. We based on Morselli and Passini (2018) and Van Hiel and Mervielde (2003) to measure participants’ interest on politics. We asked participants to answer the following four items: “How important is politics in your life?” (From 1, *nothing important*, to 5, *very important*); “How often do you talk about politics with your friends?” (From 1, *never*, to 5, *many times*); “To what extent are you interested in politics?” (From 1, *nothing*, to 5, *a lot*) and “How often do you consult political news in the media?” (1, *never*, 5, *many times*). We calculated an average of all items to use a single measure of interest in politics (α = .90) in which higher scores reflected greater interest in politics.

# Supplementary Analysis

**Supplementary Table 3.** Multivariate analysis for the effect of type of sample (students’ parents, bus station and social network) on the variables included in Study 1

|  | *F*_(2,181)_ | *p* | $\eta_{p}^{2}$ |
| --- | --- | --- | --- |
| Economic crisis (perceived threat) | 2.28 | .105 | .03 |
| Socioeconomic Status (SES) | 2.21 | .113 | .02 |
| Dangerous worldview | 2.38 | .096 | .03 |
| Authoritarianism | 0.61 | .545 | .007 |
| Trust in authoritarian leader | 1.95 | .145 | .02 |

**Supplementary Table 4.** Hierarchical regression analysis for the effect of dangerous worldview, socio-political control and covariates on authoritarianism.

|  | **β** | ***t*** | ***p*** |
| --- | --- | --- | --- |
| **Model 1** | *R*^2^-chg = .03, *F*(1, 388) = 13.56, *p* < .001 | | |
| Dangerous worldview | .18 | 3.68 | < .001 |
| **Model 2** | *R*^2^-chg = .03, *F*(1, 387) = 12.39, *p* < .001 | | |
| Dangerous worldview | .16 | 3.24 | .001 |
| Sociopolitical control | -.18 | -3.52 | < .001 |
| **Model 3** | *R*^2^-chg = .04, *F*(2, 385) = 7.56, *p* = .001 | | |
| Dangerous worldview | .20 | 3.95 | < .001 |
| Sociopolitical control | -.16 | -3.22 | .001 |
| Age | .13 | 2.73 | .007 |
| Sex | .134 | 2.73 | .007 |
| **Model 4** | *R*^2^-chg = .03, *F*(1, 384) = 11.25, *p* = .001 | | |
| Dangerous worldview | .16 | -3.25 | .001 |
| Sociopolitical control | -.12 | -2.38 | .018 |
| Age | .15 | 3.08 | .002 |
| Sex | .15 | 2.99 | .003 |
| Political Interest | -.17 | -3.35 | .001 |
| **Model 5** | *R*^2^-chg = .14, *F*(1, 383) = 75.59, *p* < .001 | | |
| Dangerous worldview | .15 | 3.36 | .001 |
| Sociopolitical control | -.04 | -0.84 | .399 |
| Age | .12 | 2.71 | .007 |
| Sex | .16 | 3.58 | .001 |
| Political Interest | -.15 | -.327 | .001 |
| Political orientation | .39 | 8.69 | < .001 |

# Alternative analyses

## Interactions effect between SES and perceived threat by the economic crisis

Following the suggestions provided during the review process, we checked for interaction effects between the perceived threat from the economic crisis and participant’s SES. Specifically, we analyzed whether participant’s SES moderated the relationship between perceived threat and the mediating variables (dangerous worldview and authoritarianism). We carried out moderation analyses using PROCESS (Model 1, bootstrapping procedure, 10,000 repeats, 95% CI), considering perceived threat as predictor variable (X), dangerous worldview/authoritarianism as dependent variable (Y), and SES as moderator (W). For both studies, results did not show any statistically significant interaction effect, suggesting that the effect of perceived threat on dangerous worldview and authoritarianism are independent of the participant’s socioeconomic status.

## Indirect effect of dangerous worldview on trust in the authoritarian leader through the two components of authoritarianism.

Following the suggestions provided during the review process, we have explored which of the two authoritarianism components drives the effect of dangerous worldview on trust in the authoritarian leader. For each study, we tested a multiple mediation model (PROCESS, Model 4, bootstrapping procedure, 10,000 repeats, 95% CI) considering dangerous worldview as predictor (X), trust in the authoritarian leader as dependent variable (Y) and both Aggression and Submission as parallel mediators (M).

Regarding Study 1, results did not clearly show that one of the two components of authoritarianism drives the effects. Rather, it seems that both components are important at different moments in the process: in the first step of the analysis (path a) the vision of the dangerous world was related to authoritarian aggression but not to submission to authority; however, in the second step of the analysis (path b) authoritarian submission, and not aggression, was significantly related to trust in the authoritarian leader (Supplementary Table 5). These effects did not vary substantially when repeating the analysis including the covariates.

Relative to Study 2, the results again did not show that one component of authoritarianism was more relevant than the other. However, unlike in Study 1, in Study 2 dangerous worldview was significantly related to both authoritarian aggression and submission to authority (path a). Also, both components of authoritarianism were positively related to trust in the authoritarian leader (path b). Thus, in Study 2 both indirect effects (through aggression and submission) were statistically significant (Supplementary Table 5). Although the indirect effect through aggression was greater than the indirect effect through submission, this difference was not statistically significant (*b* = .02, *SE* = .02, CI 95% [-.0206, .0707]). We repeated the analyses to explore whether these effects remained significant after including the covariates (sex, age, political orientation and political interest). Results showed that only the indirect effect through Aggression remained statistically significant (*b* = .03, *SE* = .02, CI 95% -.0094, .1003]).The indirect effect through Submission was no longer significant when including the covariates because neither dangerous worldview was related to Submission (*b* = .05, *SE* = .03, CI 95% [.0053, .0659]) nor Submission was related to trust in the authoritarian leader (*b* = .21, *SE* = .11, CI 95% [-.0002, .4210]).

Taken together, results appear to show that both the Authoritarian Aggression and Submission to Authority components are important in linking dangerous worldview with trust in authoritarian political leaders. However, the Aggression component appears to be a more consistent and ideologically independent mediator than Submission. These exploratory results are in line with Dunwoody and Funke's claims that Aggression is the main component of Authoritarianism followed by Submission (Dunwoody & Funke, 2016).

**Supplementary Table 5.** Multiple mediation model for the indirect effect of dangerous worldview on trust in the authoritarian leader through the two components of authoritarianism

|  | Study 1  (*N* = 184) | | | | | | Study 2  (*N* =412) | | | | | |
| --- | --- | --- | --- | --- | --- | --- | --- | --- | --- | --- | --- | --- |
|  | *B* | (*SE*) | *t* | *p*-value | LL 95% CI | UL 95% CI | *B* | (*SE*) | *t* | *p*-value | LL 95% CI | UL 95% CI |
| *Direct effects* |  |  |  |  |  |  |  |  |  |  |  |  |
| Dangerous worldview on Aggression | **.15** | (.05) | 3.09 | .002 | .0540 | .2444 | **.14** | (.04) | 3.67 | < .001 | .0653 | .2163 |
| Dangerous worldview on Submission | .05 | (.04) | 1.23 | .220 | -.0302 | .1306 | **.09** | (.03) | 3.26 | .001 | .0361 | .1458 |
| Aggression on trust in the authoritarian leader | .23 | (.12) | 1.96 | .052 | -.0021 | .4642 | **.32** | (.07) | 4.35 | < .001 | .1751 | .4641 |
| Submission on trust in the authoritarian leader | **.54** | (.14) | 3.88 | < .001 | .2673 | .8194 | **.24** | (.10) | 2.40 | .017 | .0440 | .4414 |
| Dangerous worldview on trust in the authoritarian leader | .14 | (.07) | 1.91 | .057 | -.0044 | .2868 | .07 | (.05) | 1.36 | .1739 | -.0330 | .1820 |
| *Indirect effects* |  |  |  |  |  |  |  |  |  |  |  |  |
| Dangerous worldview on trust in the authoritarian leader via Aggression | **.03** | (.02) |  |  | .0021 | .0772 | **.05** | (.02) |  |  | .0161 | .0830 |
| Dangerous worldview on trust in the authoritarian leader via Submission | .03 | (.02) |  |  | -.0147 | .0750 | **.02** | (.01) |  |  | .0021 | .0512 |
| *Total effects* |  |  |  |  |  |  |  |  |  |  |  |  |
| Dangerous worldview on trust in the authoritarian leader | **.20** | (.08) | 2.64 | .009 | .0513 | .3545 | **.14** | (.06) | 2.54 | .012 | .0320 | .2511 |

Note: Unstandardized coefficients presented. Significant coefficients emphasized in bold. LL = Lower limit; UL = Upper limit; CI = Confidence Interval.

**References**

Dunwoody, P. T. & Funke, F. (2016). The Aggression-Submission-Conventionalism Scale: Testing 986 a new three factor measure of authoritarianism. Journal of Social and Political Psychology, 4(2), 987 571–600. https://doi.org/10.5964/jspp.v4i2.168

Morselli, D., & Passini, S. (2018). Exclusive and inclusive protest in Europe: Investigating values, support for democracy, and life conditions. *Journal of Community and Applied Social Psychology, 28*(3), 123-141. https://doi.org/10.1002/casp.2345

Rousseeuw, P., & Van Zomeren, B. (1990). Unmasking Multivariate Outliers and Leverage Points: Rejoinder. *Journal of the American Statistical Association, 85*(411), 648-651. https://doi.org/10.2307/2289999

Van Hiel, A., & Mervielde, I. (2003). The measurement of cognitive complexity and its relationship with political extremism. *Political Psychology, 24*(4), 781–801. https://doi.org/10.1046/j.1467-9221.2003.00354.x
